# Clinical efficacy of metformin in familial adenomatous polyposis and the effect of intestinal flora

**DOI:** 10.1186/s13023-024-03064-6

**Published:** 2024-02-25

**Authors:** Linxin Zhou, Linfu Zheng, Binbin Xu, Zhou Ye, Dazhou Li, Wen Wang

**Affiliations:** 1 Fuzong Clinical Medical College of Fujian Medical University, Fuzhou, 350025 China; 2 Department of Gastroenterology, The 900th Hospital of Joint Logistic Support Force, PLA, Fuzhou, 350025 China; 3 Oriental Hospital Affiliated to Xiamen University, No. 156 Xierhuan North Road, Fuzhou, 350025 China

**Keywords:** Familial adenomatous polyposis, Chemoprevention, Metformin, Intestinal flora

## Abstract

**Background and aims:**

Metformin has been reported to inhibit the occurrence and development of colorectal cancer (CRC) by mediating changes in intestinal flora. Studies have also indicated that the occurence of familial adenomatous polyposis (FAP) may also be associated with changes in the intestinal flora. Therefore, we investigated the efficacy and safety of metformin in treating FAP and the association with intestinal flora.

**Results:**

Compared with the baseline, the mean number and load of polyps in the areas of nanocarbon labeling and postoperative residuals in the test group were lower than those in the placebo group, while the diversity of intestinal flora species was increased. At the genus level, the relative abundance of *g_Ruminococcus* in the test group was lower than that at baseline, whereas the relative abundance of *g_Lactobacillus* was higher. These changes were statistically significant (*P* < 0.05).

**Conclusion:**

One-year metformin therapy for FAP is safe and effective, potentially mediated by modulating the intestinal flora. This study provides new insights and strategies for preventing adenomatous polyp carcinogenesis in FAP and explores possible preventive action.

## Introduction

Familial adenomatous polyposis (FAP) is mainly caused by germline mutations in the adenomatous polyposis gene on chromosome 5 [1], and is characterized by the growth of large numbers of adenomatous polyps in the colorectum during adolescence, with a risk of progression to colorectal cancer (CRC) of close to 100% without timely intervention. Somatic mutations in the adenomatous polyposis coli (*APC*) gene occur in 80% of sporadic CRC cases, and germline mutations in the *APC* gene are the main cause of FAP; thus, it is hypothesized that the mechanism of tumorigenesis in FAP may be similar to that of the vast majority of patients with sporadic CRC [2].

Accumulating evidence supports the correlation between intestinal flora and CRC development, so further research on the relationship between intestinal flora and FAP is warranted. In patients with FAP, Dejea et al. [3] discovered a relationship between the polyposis and intestinal flora, which was mainly composed of polyketide synthase-positive *Escherichia coli* (pks + *E. coli*) and enterotoxin-producing *Bacteroides fragilis* (ETBF) in colonic biofilms. Attard et al. [4] were the first to initiate a study of the intestinal flora of pediatric patients with FAP at the mucosal level. The results suggested that, in pediatric patients with FAP, the key flora components on polyp mucosa have pro-cancer properties.

Although resection of the primary site is currently the best treatment for FAP, surgery is not a complete cure. Post-surgical patients remain at risk of developing extracolonic disease. For this reason, endoscopic treatment combined with chemoprophylaxis is slowly gaining acceptance [5]. Existing chemotherapy includes nonsteroidal anti-inflammatory drugs (NSAIDS) [6–8], cyclooxygenase inhibitor combination therapy [9, 10], mammalian target of rapamycin (mTOR) inhibitors [11–13], and traditional Chinese medicine [14]. As a result of adverse effects found in some trials, the use of most of these drugs has been limited due to concerns that long-term use could lead to gastrointestinal damage or cardiovascular events [15–17].

Numerous studies have indicated that metformin has the potential to chemoprevent CRC. In 2010, a study in Japan first reported preliminary evidence that metformin inhibits the occurrence of CRC. The results indicated that metformin inhibited the proliferation of human colonic epithelium as well as the formation of rectal abnormal crypt foci (ACF) [18]. In 2016, a clinical trial suggested that low-dose metformin not only reduces the incidence, but also the number of heterochronic adenomas after polypectomy, and also demonstrated that it is safe for non-diabetic patients to take low-dose metformin for 1 year [19]. In addition, our previous study confirmed that metformin exerted a chemopreventive effect in mice, inhibiting the occurrence and development of ACF, polyps and CRC [20, 21]. In the clinic, metformin combined with endoscopy was confirmed to be superior to endoscopy alone in the treatment of FAP [22].

The effects of metformin on the intestinal flora have been reported, with some results indicating that metformin increased the abundance of short-chain fatty acid-producing and mucin-degrading bacteria [23, 24]. Metformin also promoted the enrichment of beneficial bacteria [25].

In this study, we investigated the ability of metformin to inhibit the development and progression of FAP adenomatous polyps by regulating the intestinal flora.

## Methods

### Subjects and study design

This prospective randomized controlled double-blind trial was conducted at the endoscopy center of the 900th Hospital of Joint Logistic Support Force. Twenty-six patients who attended from January 2022 to July 2022 and met the diagnostic criteria for FAP were included. The clinical diagnostic criteria for FAP required more than 100 adenomas in the general population or more than 20 in patients with a genetic predisposition. The study protocol adhered to the Declaration of Helsinki, and all participants provided written informed consent. The study was approved by the Ethics Committee of the 900th Hospital of the Joint Security Force and registered in the China Clinical Trial Registry (ChiCTR2300071081). All authors had access to the study data and reviewed and approved the final manuscript.

Inclusion criteria were as follows: 1. Patients aged 18 years or older with a clinical diagnosis or genetic test results confirming FAP. 2. Either of the following conditions sufficed: (a) Patients with an intact colon with moderate adenomatous loads (100–1000 polyps) who were being considered for prophylactic surgery. (b) Patients who had undergone surgical procedures, primarily consisting of subtotal colectomy with ileo-rectal anastomosis (IRA) and ileal-pouch-anal-anastomosis (IPAA).

Exclusion criteria included: (1) Coexisting diseases affecting the intestinal microecology, such as inflammatory bowel disease (IBD). (2) Use of immunosuppressants and glucocorticoids within 3 months, antibiotics, probiotics within 1 month, or proton-pump inhibitors, bismuth-containing drugs within 2 weeks prior to study inclusion. (3) Diagnosis of malignant diseases such as CRC. (4) Use of NSAIDs more than 3 times per week in the 6 months before the test. (5) Diagnosis of diabetes mellitus, pregnancy, or breastfeeding. (6) Significant liver and renal function test abnormalities. (7) Men preparing for conception. Other factors deemed inappropriate for enrollment by the investigator.

**Fig. 1:**
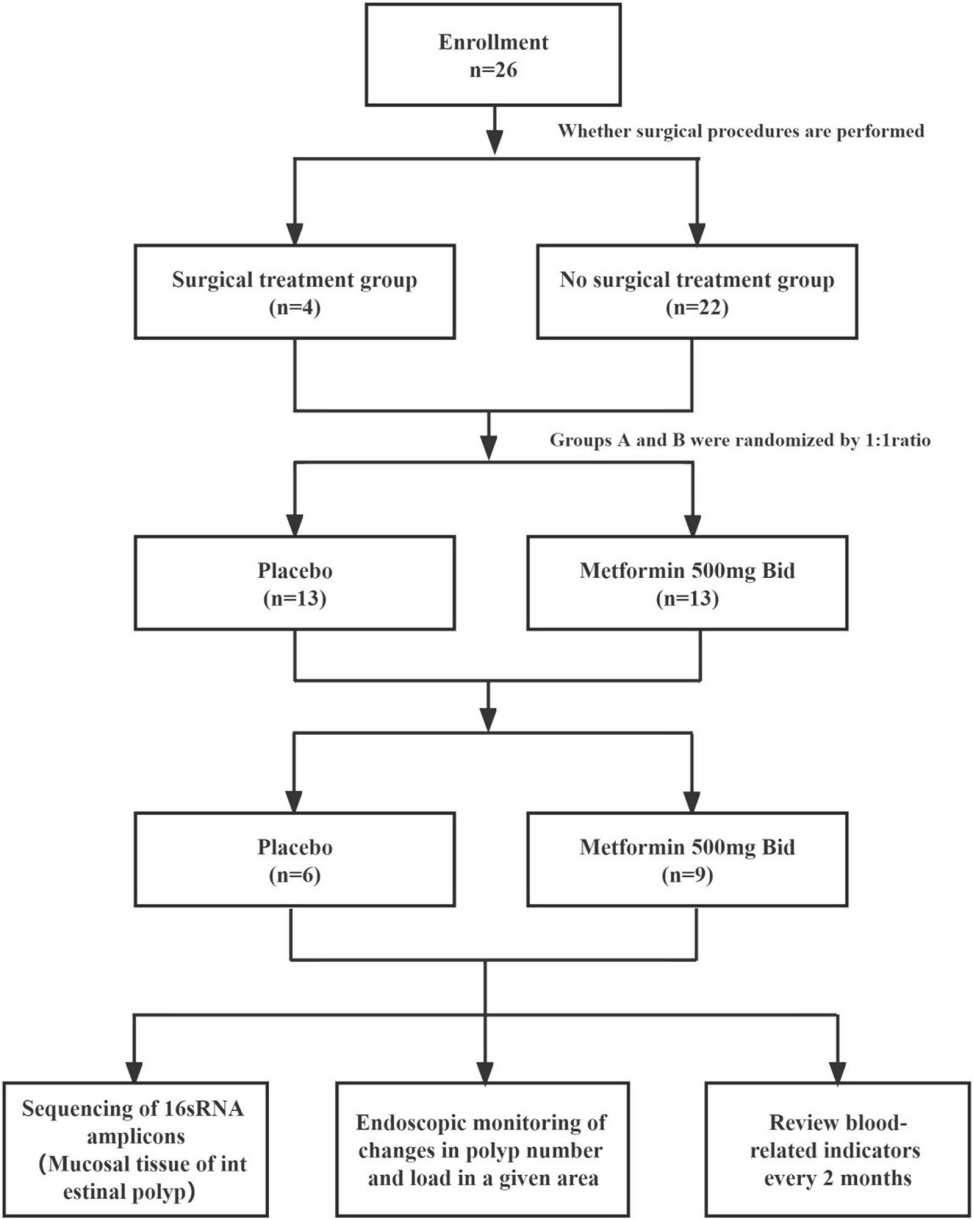
A schematic diagram of the study protocol

The study subjects were initially divided into a surgical treatment group and a non-surgical treatment group based on whether they underwent surgery. Each group was then randomly assigned in a 1:1 ratio to either a placebo group or a test group according to the order of enrollment, using a random number method, for a study period of 1 year. During the follow-up, the placebo group received a placebo matching metformin in size, appearance, and odor, primarily composed of starch. In contrast, the test group received 500 mg of metformin (produced by Tianfang Pharmaceutical Company) twice daily. Randomized grouping and medication distribution were conducted blindly by a nurse, with grouping and medication information concealed from both the managing physician and the participants until the study's conclusion. A schematic diagram of the study protocol is shown in Fig. 1.

During the baseline period, patients who had not undergo surgery received upper gastrointestinal endoscopy and colonoscopy. A 10 cm section in the middle part of the descending colon was selected for nanocarbon labeling. For patients who underwent IRA or IPAA, the remaining the rectal or ileal storage pouch area was identified for observation, depending on the surgery type. Endoscopic resection targeted all polyps more than 1 cm in diameter in the gastrointestinal tract, with immediate surgical treatment recommendation if pathology suggested a high-grade atypical hyperplasia or adenocarcinoma. After 1 year, the designated areas (including the descending duodenum, the nanocarbon-labeled area, and the postoperative residual area) were reassessed, focusing on changes in polyps numbers and load.

The endoscopy was video-recorded, with the operator and two other experienced endoscopists on-site to perform the first count of polyps in a designated area, while images were taken on-site in freeze-frame. Images were selected by two independent and experienced endoscopists to compare polyp changes before and after treatment of the designated areas for a second data review. The video served as a reference to resolve disagreements and assist in confirming the counts of polyp. The operator assessed the polyp size by closing and opening the biopsy forceps, only polyps with a diameter of ≥ 2 mm were included during the count. We categorized polyp diameters into the following five categories: 2–4 mm, 5–6 mm, 7–8 mm, 9–10 mm, and > 10 mm, and multiplied the number of polyps in each category by the median number of polyp diameters corresponding to that category. Finally, all the polyp diameters were summed to obtain the polyp load. Polyp mucosal tissue was also obtained from areas that were not counted.

### Tissue processing and bioinformatics analysis

Intestinal polyp mucosal tissues were collected from both groups at baseline and at endoscopy after 1 year, processed with liquid nitrogen and immediately frozen and stored in an ultra-low-temperature refrigerator at – 80 °C. Primers (515F and 806R) targeting the V4 region of the bacterial 16S rRNA gene were used. Paired-end reads were assigned to samples based on their unique barcode and truncated by cutting off the barcode and primer sequence. The valid data were then rigorously processed to obtain the final amplicon sequence variants (ASVs) using the DADA2 module in the QIIME2 software (Version QIIME2-202006) for noise reduction, while ɑ and β diversity were calculated using QIIME2.

### Statistical analysis

All statistical analysis was performed using SPSS 25.0 software. Normally distributed continuous variables are presented as mean ± standard deviation (SD), and comparisons between the two groups were conducted using either the two independent samples t-test or the paired samples t-test. Non-normally distributed continuous variables were expressed as median (quartiles), and comparisons between the two groups were performed using the Mann–Whitney rank sum test. The level of significance was set at α = 0.05, and the test was statistically significant (*P* < 0.05).

### Primary and secondary end-points

Primary end-points: Changes in the number of adenomatous polyps in the designated area recorded before and after the trial were compared first, by comparing the two groups and second, by comparing the changes in the load of adenomatous polyps.

Secondary end-points: Changes in the composition of the intestinal flora and the relative abundance of species in the two groups recorded before and after the trial.

### Assessment of drug safety

Patient adherence and monitoring of adverse events were assessed by conducting monthly telephone follow-up, micro-telephone video, or in-person conversations. To evaluate the safety of metformin, a range of blood tests were conducted every 2 months. These tests included fasting blood glucose (FBG), fasting insulin (FI), glycosylated hemoglobin (HbA1c), aspartate aminotransferase (AST), alanine aminotransferase (ALT), blood urea nitrogen (BUN), blood creatinine, total cholesterol (TC), and low-density lipoprotein (LDL). Adverse events were systematically graded using the Common Terminology Criteria for Adverse Events, version 4.0 (CTCAE v4.0).

## Results

### Baseline characteristics of the study population

Of the 26 FAP patients who met the enrollment criteria, seven dropped out of the placebo group and four dropped out of the test group. Of the remaining 15 patients, eight participated in genetic testing and had results suggestive of mutations in the *APC* gene. F1 and F2 were sons of F10, F3 was a nephew of F10, F4 was a son of F12, F5 and F7 were sisters, and F6 was a daughter of F7. Four patients underwent surgery, three patients retained their smoking habit to date, and two patients presented with extracolonic manifestations of which one patient presented with congenital hypertrophy of the retinal pigment epithelium (CHRPE) and desmoid tumor (DT), and the other patient presented with DT. Baseline demographic data characteristics are shown in Table 1.

**Table 1:** Baseline demographic data characteristics of patients

NO	Sex	Age	Family history	APC mutation	Operation	EIM	Current smoker
1	M	32	Mother	Exon16 (codon 1062)	NO	NO	Yes
2	M	30	Mother	Exon16 (codon 1062))	NO	NO	Yes
3	M	21	Father	Exon16 (codon 1062)	NO	NO	NO
4	M	18	Father	N/A	NO	NO	NO
5	F	66	Father	Exon15 (codon 716)	Yes	CHRPE、DT	NO
6	F	24	Mother	Exon16 (codon 690)	NO	NO	NO
7	F	59	Father	Exon16 (codon 690)	NO	NO	NO
8	M	18	Father	Exon17 (codon 1114)	NO	NO	NO
9	M	65	unknown	N/A	NO	NO	NO
10	F	51	Mother	Exon16 (codon 1062)	Yes	NO	NO
11	M	71	Father	N/A	NO	NO	Yes
12	M	44	Mother	N/A	Yes	DT	NO
13	M	19	Father	N/A	NO	NO	NO
14	M	57	Mother	N/A	Yes	NO	NO
15	F	32	Mother	N/A	NO	NO	NO

N/A, not available; EIM, extraintestinal manifestation; CHRPE, congenital hypertrophy of the retinal pigment epithelium; DT, Desmoid tumor

### Primary end-point results

At the baseline level, there was no significant difference between the placebo group and the test group in the mean number and load of polyps in the designated areas (*P* > 0.05). After 1 year, there was still no significant difference between the two groups in the mean number and load of polyps in the descending part of the duodenum (*P* > 0.05). In contrast, the mean number and load of polyps in the area of nanocarbon-labeling and residual area after the operation were reduced in the test group compared with those in the placebo group (*P* < 0.05).

**Fig. 2:**
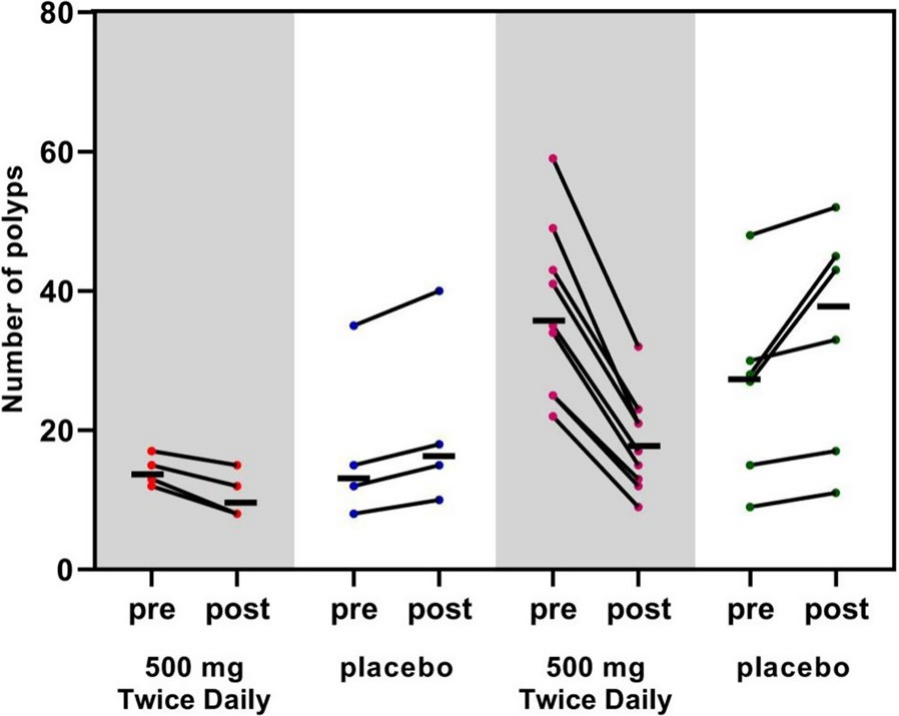
The number of polyps changes in Schematic diagram. Mean number of polyps (indicated by horizontal lines). The two groups on the left indicate changes in the number of polyps in the descending duodenal region; the two groups on the right indicate changes in the number of polyps in the nanocarbon-labeled and postoperative residual regions

**Fig. 3:**
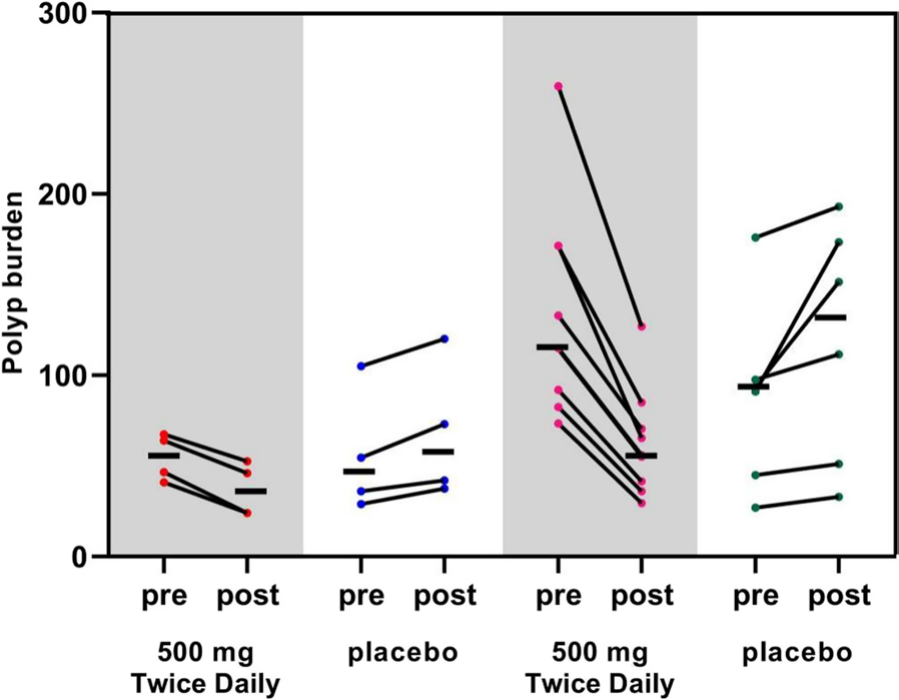
Polyps burden changes in Schematic diagram. Mean polyp load (indicated by horizontal line) in millimeters (mm). The two groups on the left indicate changes in polyp load in the descending duodenal region; the two groups on the right indicate changes in polyp load in the nanocarbon-labeled and postoperative residual regions

After 1 year, the mean number of polyps in the designated area of the placebo group had increased from the number detected at the baseline (*P* < 0.05), whereas the mean number in the test group had decreased (*P* < 0.05). The difference in the change in the mean number of polyps in the designated areas between the two groups was statistically significant compared with the baseline after 1 year (*P* < 0.001) (Fig. 2 and Table 2). After 1 year, the mean polyp load in the placebo group had increased from that detected at the baseline in the descending duodenum (*P* = 0.013) and in the nanocarbon-labeled and post-surgical residual regions (*P* = 0.066). In contrast, the mean polyp load in the test group had decreased significantly from that detected at the baseline in the indicated regions (*P* < 0.001). After 1 year, the difference in the change in the mean polyp load of the two groups in the designated region was statistically significant when compared to that detected at baseline (*P* < 0.001) (Fig. 3 and Table 3).

**Table 2:** Effect of metformin on the number of polyps at different segments

Segment	Number of polyps at baseline	Number of polyps at 1 year	*p* value
*Descending duodenum*			
Test group	14.25 ± 2.22	10.75 ± 3.40	0.012
Placebo	17.5 ± 12.01	20.75 ± 13.25	0.014
*p* value	0.077	0.109	
*Carbon nanoparticles tattooing and Post-surgical residual areas*			
Test group	37.00 ± 12.28	18.11 ± 6.99	< 0.001
Placebo	26.17 ± 13.53	30.67 ± 14.71	0.012
*p* value	0.132	0.043

**Table 3:** Effect of metformin on polyp loading at different segments

Segment		Number of polyps at baseline	Number of polyps at 1 year	*p* value
Descending duodenum	Test group	54.75 ± 12.98	36.63 ± 14.82	< 0.001
	Placebo	56.13 ± 34.30	68.88 ± 37.35	0.013
	*p* value	0.943	0.160	
Carbon nanoparticles tattooing and Post-surgical residual areas	Test group	134.72 ± 58.46	62.89 ± 29.73	< 0.001
	Placebo	88.33 ± 51.85	118.92 ± 65.69	0.066
	*p* value	0.140	0.041	

### Secondary end-point results

#### Cluster analysis of ASVs in the intestinal flora

**Fig. 4:**
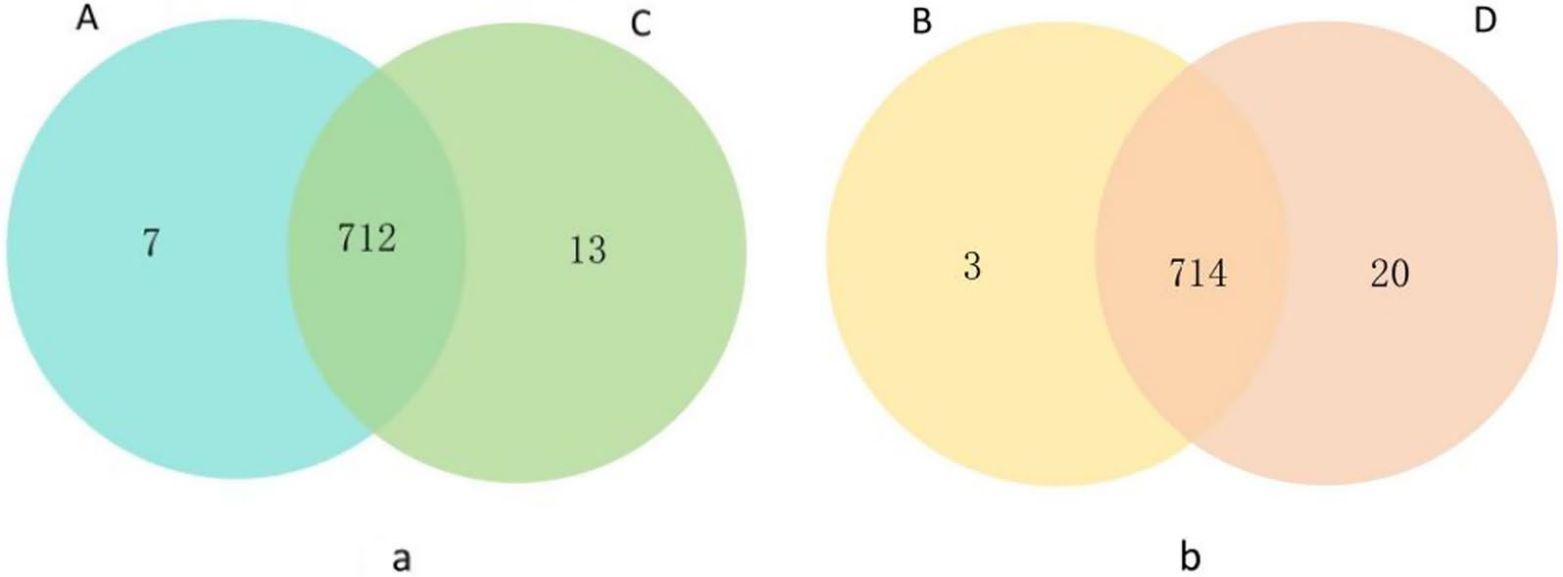
Venn diagram. *Note*: Blue circles represent baseline levels in the placebo group, green circles represent baseline levels in the test group, yellow circles represent after 1 year in the placebo group, and orange circles represent after 1 year in the test group. Overlapping portions of different circles indicate ASVs sequences common to both groups. The numbers labeled in the figure represent the number of ASVs sequences in different sections

At the baseline level, 719 ASVs were identified in the placebo group (A) and 725 in test group (C). Of these ASVs, 712 were shared by the two groups, while seven were unique to the placebo group and 13 were unique to the test group (Fig. 4a). After 1 year, 717 ASVs were identified in the placebo group (B) and 734 in the test group (D). Of these ASVs, 714 were shared by the two groups, while three were unique to the placebo group and 20 were unique to the test group (Fig. 4b). These data revealed that metformin intervention did not significantly increase the number of ASVs.

#### Relative abundance of species in the intestinal flora

**Fig. 5:**
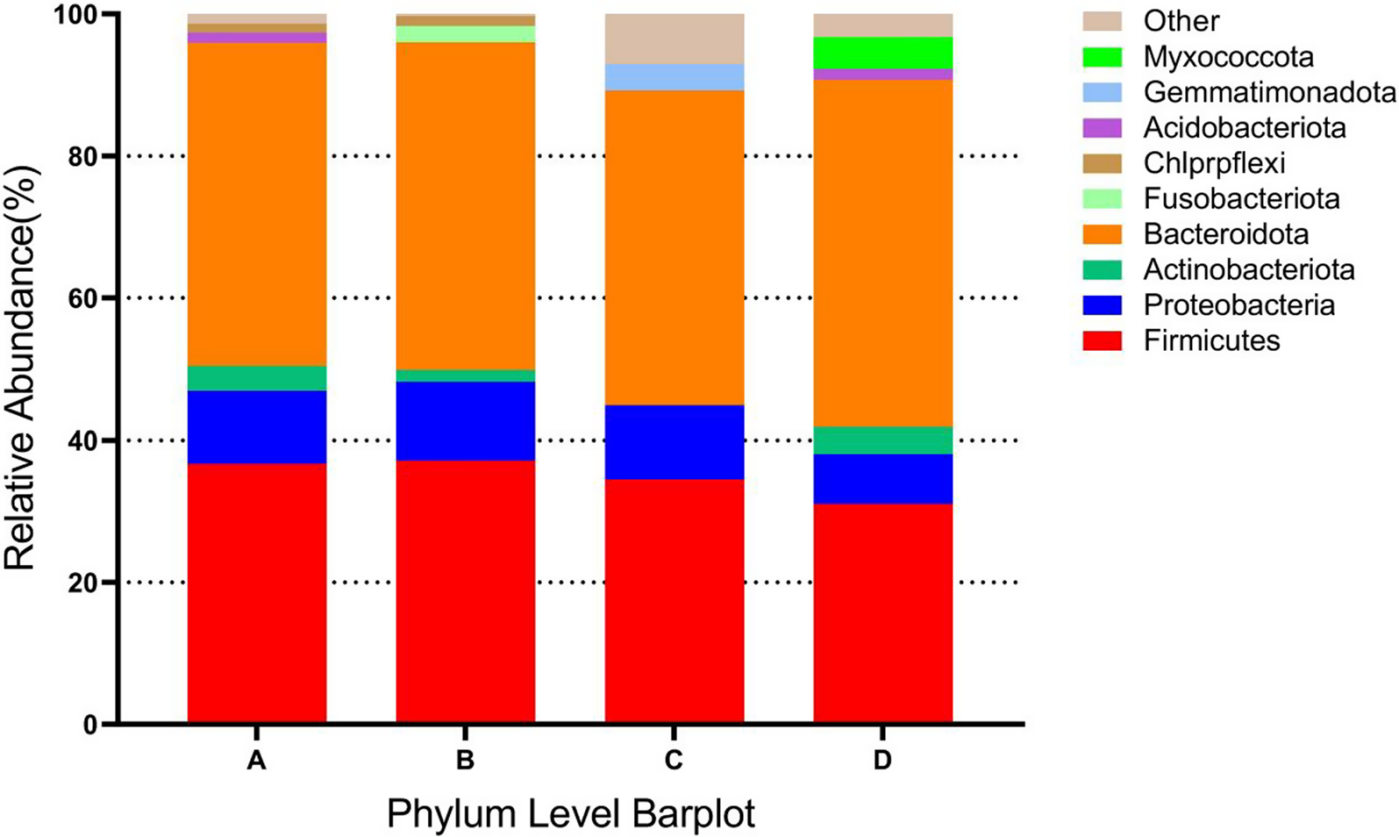
Relative abundance of species at the phylum level. Panel **A** represents the baseline level of the placebo group, panel **B** represents the placebo group after 1 year, panel **C** represents the baseline level of the test group, and panel **D** represents the test group after 1 year

**Fig. 6:**
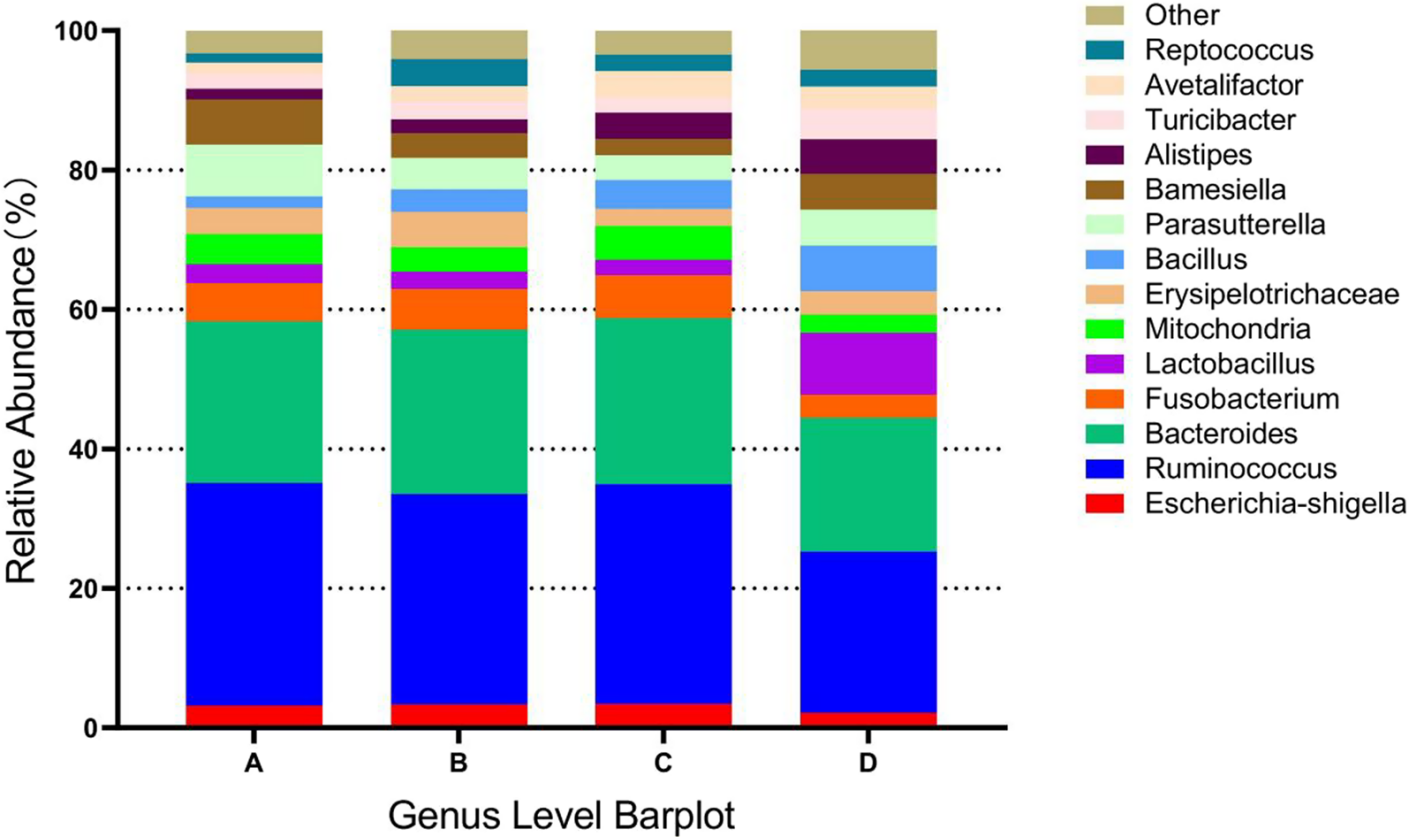
Relative abundance of species at the genus level. Panel **A** represents the baseline level of the placebo group, panel **B** represents the placebo group after 1 year, panel **C** represents the baseline level of the test group, and panel **D** represents the test group after 1 year

Based on the results of species annotation, the top 10 species with highest abundance at the phylum level for each subgroup were selected to generate the bar chart of species relative abundance shown in Fig. 5. *Bacteroidetes*, *Firmicutes*, and *Proteobacteria* accounted for the highest percentages of species in the two groups. Futhermore, the top 15 species with highest abundance at the genus level for each subgroup were selected to generate the bar chart of species relative abundance shown in Fig. 6. After 1 year of metformin administration, the relative abundance of *g_Ruminococcus* in the test group was lower than that at the baseline (23.14% vs. 31.46%, *P* < 0.05), whereas the relative abundance of *g_Lactobacillus* was higher than that at the baseline (8.91% vs. 2.26%, *P* < 0.05). Additionally, the relative abundances of *g_Escherichia-shigella*, *g_Bacteroides*, and *g_Fusobacterium* in the test group were lower than those at the baseline level (*P* > 0.05).

#### Alpha diversity analysis of the intestinal flora

Analysis of the differences in the gut flora ɑ diversity index between the two groups (Table 4) suggested that the abundance as well as the diversity of gut flora species in the test group increased after 1 year of metformin administration compared to the baseline. This was evidenced by significantly higher values of Shannon’s index (*P* = 0.025) and Simpson’s index (*P* = 0.009) than those at the baseline.

**Table 4:** Alpha diversity analysis of the gut microbiota

	Observed species	Chao1	Shannon	Simpson
*At baseline*				
Placebo	822	405.3	6.614	0.902
Test group	810	420.7	6.098	0.908
*p* value	0.596	0.906	0.456	0.480
*At 1 year*				
Placebo	806	411	6.516	0.888
Test group	863	443	7.392	0.989
*p* value	0.289	0.814	0.025	0.009

#### Beta diversity analysis of the intestinal flora

**Fig. 7:**
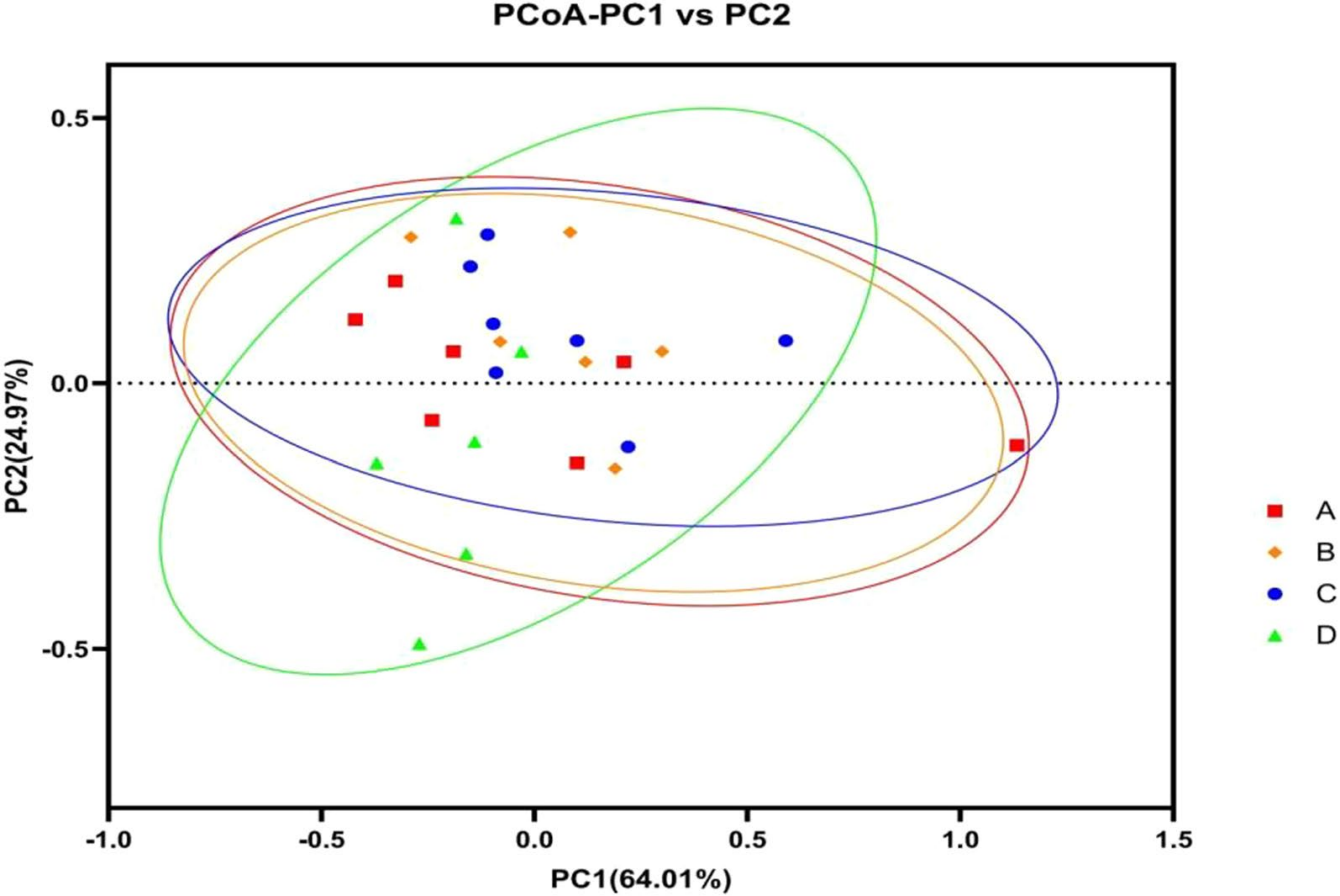
Beta diversity analysis of intestinal flora

Weighted Unifrac Principal Coordinates Analysis (PCoA) analysis was performed to compare the species composition of the intestinal flora in FAP patients in both groups at baseline and after 1 year. The analysis revealed no significant trend of separation along PC1 or PC2, indicating a similarity in species composition. The horizontal coordinate (PC1) explained 64.01% of the differences, while the vertical coordinate (PC2) accounted for 24.97%. According to the analysis of similarities (ANOSIM), there was no significant difference in the species composition of the intestinal flora between the two groups (*P* > 0.05) (Fig. 7).

#### Drug safety assessment of metformin

The differences in blood creatinine, BUN, TC, LDL, AST, ALT, HbA1c, body mass index (BMI), and homeostatic model assessment of insulin resistance (HOMA-IR) between the two groups were analyzed at baseline and after 1 year (Table 5). In the test group, there were no significant changes in these indexes after 1 year of metformin treatment compared to the baseline measurements (*P* > 0.05), with the HOMA-IR remaining constant during this period. Adverse events occurred in three patients (23.1%), all of which were classified as NCI-CTCAE grade 1, indicating mild severity (Table 6). These included abdominal pain, diarrhea, and rash. Notably, One patient withdrew from the study due to an adverse event (diarrhea).

**Table 5:** Drug safety assessment of metformin

	Test group	Placebo	*p* value
*Creatinine (mg/dL)*			
At baseline	72.92	67.73	0.126
At 1 year	72.5	69.22	0.352
*p* value (baseline vs. 1 year)	0.913	0.378	
*Blood urea nitrogen (mg/dL)*			
At baseline	3.39	4.92	0.02
At 1 year	3.73	5.42	0.006
*p* value (baseline vs. 1 year)	0.229	0.575	
*Total cholesterol (mg/dL)*			
At baseline	3.75	2.93	0.112
At 1 year	3.74	2.62	0.023
*p* value (baseline vs. 1 year)	0.978	0.66	
*LDL cholesterol (mg/dL)*			
At baseline	2.06	2.26	0.768
At 1 year	2.21	2.34	0.988
*p* value (baseline *vs*1 year)	0.757	0.735	
*Aspartate aminotransferase (AST)*			
At baseline	19.6	14.85	0.045
At 1 year	17.4	15.22	0.061
*p* value (baseline vs. 1 year)	0.377	0.886	
*Alanine aminotransferase (ALT)*			
At baseline	33.19	15.02	0.089
At 1 year	18.29	16.47	0.336
*p* value (baseline vs. 1 year)	0.008	0.395	
*HbA1c (%)*			
At baseline	5.53	5.45	0.494
At 1 year	5.49	5.55	0.558
*p* value (baseline vs. 1 year)	0.665	0.412	
*HOMA-IR*			
At baseline	1.18	0.97	0.528
At 1 year	1.16	0.93	0.335
*p* value (baseline vs. 1 year)	0.968	0.826	
*BMI (Kg/m*^*2*^*)*			
At baseline	23.10	21.7	0.491
At 1 year	22.16	21.57	0.745
*p* value (baseline vs. 1 year)	0.543	0.956	

Data are mean (SD). HOMA = fasting blood × glucose fasting insulin/22.5

**Table 6:** Adverse events

	Test group (n = 13)	Placebo (n = 13)
Abdominal pain	1 (7.7%)	0
Diarrhea	1 (7.7%)	0
Rash	1 (7.7%)	0

## Discussion

Many clinical trials have shown that chemopreventive drugs can inhibit the growth of adenomatous polyps in patients with FAP. However, their long-term use is often limited by side effects. Metformin, a well-known first-line antidiabetic drug, has also been recognized for its antitumor effects [26–28].

Previous studies have indicated that certain bacteria may promote the occurrence and development of CRC. Metformin is thought to exert its preventive and therapeutic effects on CRC by affecting the intestinal flora [29–31]. As research progresses, the significance of changes in the intestinal flora of FAP patients is becoming increasingly acknowledged. Dejea et al. reported that co-colonization by pks + *E. coli* and ETBF promoted the development and progression of cancer in FAP patients [3]. Futhermore, Kim et al. [32] observed that FAP patients had a significantly lower diversity of intestinal flora compared to healthy individuals, and also a higher ratio of thick-walled phyla to anaplasmatoid phyla, as well as greater relative abundance of metaplasmatoid phyla. Based on these finding, our hypothesis is that metformin could potentially inhibit the occurrence and development of adenomatous polyps in FAP by regulating the intestinal flora.

In this study, we found that after 1 year of metformin treatment, the mean number of polyps and the mean polyp load in the designated areas were significantly reduced in the test group compared with baseline (*P* < 0.05). Futhermore, both the mean number of polyps and the mean polyp load in the nanocarbon-labeled and postoperative residual areas were much lower in the test group than in the placebo group (*P* < 0.05). These results suggest that metformin may inhibit the incidence and development of adenomatous polyps in FAP patients. Our findings align with those of Higurashi et al., who reported that low-dose metformin administered over 1 year suppressed the development of heterochronic adenomas after polypectomy [19]. Conversely, a Korean study in randomized, double-blind clinical trial found no significant reduction in the mean number and size of colorectal or duodenal adenomas in FAP patients after 7 months of metformin treatment at two different doses [33]. The shorter duration of this trial could be a key reason for the observed, lack of adenoma regression in FAP patients, who may need a longer treatment period to see the effects similar to those in most cases of sporadic adenomas. Additionally, the small sample size of this trial may explain the limited clinical impact of the modest reductions in polyps number and size observed in FAP patients.

The flora assays conducted in this study indicated an increased diversity of the intestinal flora following metformin treatment compared to the baseline. Specifically, after 1 year of metformin administration, there was an increase in the relative abundance of *Bacteroidetes*, and a decrease in *Firmicutes* and *Proteobacteria*. In animal experiments, Liu et al. [34] found that the relative abundance of *Bacteroidetes* in the feces of the metformin-treated group increased compared with that of the normal control group, whereas the relative abundance of Firmicutes decreased. In a clinical trial, Yuan et al. [35] found that the relative abundance of *Proteobacteria* and *Bacteroidetes* in the feces of metformin-treated group increased, while the relative abundance of Firmicutes decreased in the metformin-treated group. These patterns mirror the changes in *Firmicutes* and *Bacteroidetes* seen in our study.

Morever, after 1 year of metformin administration, the relative abundances of *g_Escherichia-shigella*, *g_Ruminococcus*, *g_Bacteroides*, and *g_Fusobacterium* in the test group decreased from the baseline, whereas the relative abundance of *g_Lactobacillus* increased. Liu et al. [36] also found that metformin restored the biodiversity of the intestinal flora in IBD mice, leading to a decreased abundance of* g_Escherichia-shigella* and increased abundances of *g_Lactobacillus,* and *g_Akkermansia*. Additionally, it was reported that metformin significantly reduced the relative abundance of *g_Bacteroides* in the feces of mice and inhibited CRC development [31].

Futhermore, several studies have indicated that *Fusobacterium nucleatum* (*F. nucleatum*) could promote the occurrence and development of CRC [37–40], while metformin has been shown to alleviate symptoms and inhibit the formation of intestinal tumors in *F. nucleatum*-administered APCMin/ + mice [28]. Additionally, *g_Ruminococcus* has been increasingly recognized for its connection to metabolic diseases [41]. This evidence collectively suggests that metformin may contribute to restoring species diversity in the intestinal flora and inhibits the development and progression of adenomatous polyps in patients with FAP by altering the relative abundance of specific genera.

However, this study has several limitations. Firstly, the limited sample size, comprising a small number of families, may not fully capture the chemopreventive effect of metformin in different groups. Secondly, regarding the design of the study endpoints, we focused only on the changes induced by metformin on polyp number and load. For FAP chemoprevention, more critical endpoints might include delaying major surgery, endoscopic resection of advanced adenomas, the emergence of highly atypical hyperplasia in rectal or ileal reservoirs, and duodenal disease progression [42]. The relatively brief duration of our study also limits insights into the long-term impacts of metformin on FAP patients. Lastly, the absence of fecal collection in our study precluded a comparative analysis of metformin's effects on mucosal and fecal flora. Future studies, incorporating a larger cohort and extended trial periods, are essential to more definitively ascertain metformin's chemopreventive efficacy in FAP patients.

## Conclusion

We conclude that 1-year metformin therapy for FAP is both safe and effective, potentially exerting its effects by modulating the intestinal flora. This study not only offers novel insights and approaches for preventing adenomatous polyp carcinogenesis in FAP, but also sheds light on the possible mechanisms underlying such preventive actions.

## Data Availability

All data generated or analysed during this study are included in this published article [and its supplementary information files].

## Abbreviations

FAP: Familial adenomatous polyposis
CRC: Colorectal cancer
APC: Adenomatous polyposis coli
mTOR: Mammalian target of rapamycin
pks + *E. coli*: Polyketide synthase-positive *Escherichia coli*
ETBF: Enterotoxin-producing *Bacteroides fragilis*
ACF: Rectal abnormal crypt foci
NSAIDS: Nonsteroidal anti-inflammatory drugs
IBD: Inflammatory bowel disease
IRA: Ileo-rectal anastomosis
IPAA: Ileal-pouch-anal-anastomosis
ASVs: Amplicon sequence variants
SD: Standard deviation
FBG: Fasting blood glucose
FI: Fasting insulin
HbA1c: Glycosylated hemoglobin
BMI: Body mass index
AST: Aspartate aminotransferase
ALT: Alanine aminotransferase
BUN: Blood urea nitrogen
TC: Total cholesterol
LDL: Low-density lipoprotein
HOMA-IR: Homeostatic model assessment of insulin resistance
CHRPE: Congenital hypertrophy of the retinal pigment epithelium
DT: Desmoid tumor
PCoA: Rincipal coordinates analysis
ANOSIM: Analysis of similarities
*F. nucleatum*: *Fusobacterium nucleatum*
